# Alverine-Loaded Lipid Bilayer–Graphene Oxide Hybrids as a Novel Nanomedicine Platform for Neural Cancer

**DOI:** 10.3390/ijms27073273

**Published:** 2026-04-04

**Authors:** Alicja Przybylska, Irina Naletova, Francesco Attanasio, Katarzyna Dopierała, Agnieszka Kołodziejczak-Radzimska, Cristina Satriano

**Affiliations:** 1 Institute of Chemical Technology and Engineering, Poznan University of Technology, Berdychowo 4, 60-965 Poznan, Poland; 2 Institute of Crystallography, National Council of Research (IC-CNR), via Paolo Gaifami, 18, 95126 Catania, Italy; 3 Nano Hybrid Biointerfaces Laboratory (NHBIL), Department of Chemical Sciences, University of Catania, viale Andrea Doria, 6, 95125 Catania, Italy

**Keywords:** 2D materials, lipid vesicles, drug repurposing, neuroblastoma, SH-SY5Y, confocal microscopy, fluorescence imaging

## Abstract

Graphene oxide (GO)–lipid hybrid nanostructures represent a promising class of multifunctional platforms for drug delivery and fluorescence-guided cellular imaging. In this study, we developed a graphene oxide-supported lipid bilayer system composed of rhodamine-labeled phosphatidylcholine (POPC-Rhod) for the delivery of the repurposed antispasmodic drug alverine citrate (ALV) to neuroblastoma cells. The hybrid nanostructures were assembled using two drug-loading strategies and characterized by UV–Vis spectroscopy, fluorescence analysis, dynamic light scattering, and atomic force microscopy to evaluate molecular interactions, vesicle size distribution, and nanomechanical properties. In vitro studies were performed using human neuroblastoma SH-SY5Y cells and their retinoic acid-differentiated neuronal-like counterparts. Confocal microscopy confirmed efficient cellular uptake of the fluorescent lipid–graphene hybrids, while viability and mitochondrial reactive oxygen species assays revealed differentiation-dependent cellular responses. ALV-loaded hybrids induced cytotoxic effects in proliferating neuroblastoma cells, whereas differentiated neuron-like cells exhibited greater tolerance and, at moderate concentrations, preserved viability despite increased oxidative stress. These findings demonstrate that graphene oxide–lipid hybrids can act as fluorescence-traceable drug delivery platforms and highlight the potential of alverine as a candidate for repurposing in neural cancer models. The system presented here provides a proof-of-concept framework for the development of multifunctional nanocarriers integrating therapeutic delivery with imaging capabilities.

## 1. Introduction

Neuroblastoma remains one of the most challenging pediatric solid tumors, with high clinical heterogeneity, ranging from spontaneous regression to highly aggressive metastatic disease [1]. Despite advances in multimodal treatment strategies, including surgery, chemotherapy, radiotherapy, and immunotherapy, survival rates for high-risk neuroblastoma remain unsatisfactory [2]. This underscores the urgent need for novel therapeutic approaches and improved drug delivery strategies capable of enhancing efficacy while reducing systemic toxicity. Efforts to improve therapeutic efficacy have increasingly turned toward drug repurposing strategies and the development of nanotechnology-enabled delivery systems. Drug repurposing offers the advantage of reduced development time and established safety profiles [3], while nanocarriers can enhance intracellular delivery and improve pharmacokinetics, while minimizing off-target toxicity [4].

Alverine citrate (ALV), a phenylpropylamine derivative clinically used as an antispasmodic for gastrointestinal disorders such as irritable bowel syndrome and dysmenorrhea [5], has recently attracted attention for its potential anticancer properties [5,6,7]. Although originally developed for smooth muscle relaxation, recent evidence has suggested additional anticancer potential [8]. Specifically, emerging studies suggest that ALV may modulate apoptosis-related pathways and enhance the cytotoxic activity of proteasome inhibitors [6]. In particular, alverine has been reported to potentiate the effects of the proteasome inhibitor MG132, possibly through interference with lipid metabolism or stress-response pathways [9]. In addition to these effects, ALV acts as an antagonist of the serotonin 5-HT1A receptor [10,11].

Neuroblastoma cells are known to express serotonergic receptors, including 5-HT1A [12,13], and serotonin signaling has been implicated in tumor growth, survival, and differentiation in neural-derived cancers [14,15]. These observations provide a mechanistic rationale for exploring alverine as a repurposed therapeutic agent in neuroblastoma models [8,16], where modulation of serotonin-associated pathways may influence tumor cell behavior [17].

Nanotechnology-based drug delivery systems offer a powerful means to improve the therapeutic performance of repurposed drugs, particularly in tumors with complex microenvironments such as neuroblastoma [18], by enhancing cellular uptake, protecting labile compounds, and enabling controlled or targeted release.

Among the various nanomaterials investigated, graphene oxide (GO) has emerged as a promising platform for biomedical applications due to its large specific surface area, abundance of oxygen-containing functional groups, and intrinsic optical properties [19,20]. These features enable efficient adsorption of aromatic and hydrophobic drugs, facilitate high loading capacity, and support controlled or stimulus-responsive release profiles. In addition, GO exhibits strong near-infrared absorbance, making it suitable for imaging and photothermal applications [21].

Graphene-based nanoplatforms have been widely explored for cancer theranostics, combining drug delivery, imaging, and therapeutic functionalities within a single material. Importantly, GO nanosheets interact strongly with biological membranes and are readily internalized by cells through endocytic pathways. Exposure to GO has been associated with modulation of intracellular redox balance, mitochondrial dysfunction, and activation of stress-related signaling pathways in several cancer cell types [22,23,24,25].

These intrinsic biological effects may synergize with chemotherapeutic or repurposed drugs to enhance anticancer efficacy.

The integration of GO with lipid-based nanostructures, such as liposomes or supported lipid bilayers, offers additional advantages by combining the high loading capacity and structural stability of graphene with the biocompatibility and membrane affinity of phospholipids [26,27]. Such hybrid systems can improve colloidal stability, facilitate drug encapsulation within lipid domains, and provide a more biomimetic interface for interaction with cellular membranes.

Fluorescently labeled phospholipids provide an additional advantage by enabling direct visualization of nanocarrier internalization and intracellular trafficking.

Rhodamine-labeled phosphatidylcholine (POPC-Rhod) derivatives, in particular, are widely used as membrane probes due to their strong fluorescence and compatibility with confocal microscopy. Incorporation of such fluorophores into lipid–graphene hybrids creates systems that can simultaneously deliver therapeutic cargo and be tracked within cells, representing a key step toward multifunctional nanomedicine platforms.

While graphene-based nanocarriers and lipid–graphene hybrid systems have been explored individually for drug delivery and imaging applications, their integration with repurposed neuromodulatory drugs remains insufficiently investigated, particularly in the context of pediatric neural tumors. Moreover, the combined impact of graphene oxide–lipid hybrids and serotonin-modulating agents on neuroblastoma cell behavior, mitochondrial stress responses, and phenotype-dependent cellular uptake has not been systematically evaluated.

The present study aims to develop and evaluate a graphene oxide-supported lipid bilayer system [28] loaded with alverine as a model fluorescence-traceable nanocarrier for neural cancer applications. The platform uniquely combines (i) the high surface area and intrinsic bioactivity of graphene oxide, (ii) the membrane-mimetic and drug-encapsulating properties of phospholipid vesicles, and (iii) the pharmacological activity of a clinically approved serotonin receptor antagonist. This design enables simultaneous drug delivery, intracellular tracking, and assessment of graphene-induced cellular stress, thereby providing a multifunctional theranostic framework. We hypothesized that: (i) incorporation of ALV into lipid–graphene hybrids would enhance cellular uptake relative to free drug or liposomes alone due to graphene-mediated membrane interactions; (ii) the intrinsic oxidative and proteotoxic stress induced by graphene oxide would synergize with ALV-mediated signaling perturbation to amplify cytotoxic responses in neuroblastoma cells; and (iii) these effects would differ between proliferative and neuron-like differentiated phenotypes, reflecting variations in membrane composition, endocytic activity, and serotonergic signaling.

To test these hypotheses, rhodamine-labeled phospholipid vesicles were assembled onto ultrasonicated graphene oxide nanosheets to form POPC-Rhod@GO nanostructures using two drug-loading strategies.

The hybrids were comprehensively characterized using spectroscopic, nanomechanical, and interfacial techniques, and their biological activity was evaluated in vitro in undifferentiated and retinoic acid-differentiated SH-SY5Y neuroblastoma cells [29], to investigate uptake, cytotoxicity, and oxidative stress responses in a phenotype-dependent manner. By integrating physicochemical characterization with functional cellular assays, this work aims to elucidate the mechanistic interplay between graphene oxide, lipid membranes, and a repurposed serotonergic drug, and to establish a foundation for the rational design of multifunctional nanocarriers for neural cancer therapy.

## 2. Results

### 2.1. Fabrication and Physicochemical Characterization of ALV-Loaded POPC-Rhod@GO Hybrids

#### 2.1.1. Spectroscopic Characterization of Individual Components

**Figure 1:**
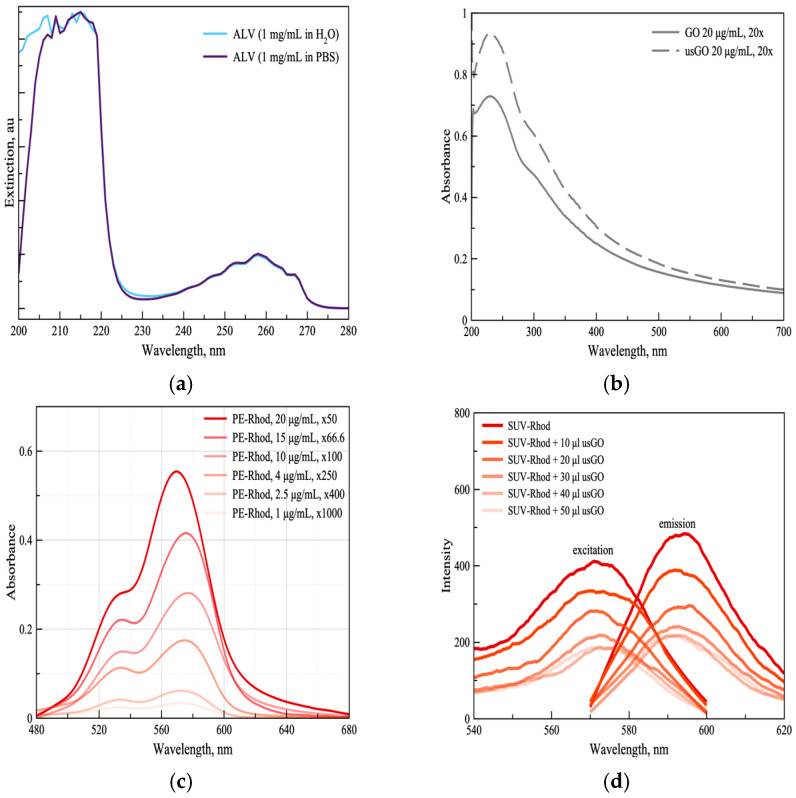
Optical characterization of the individual components used for hybrid nanostructure fabrication and spectroscopic characterization of lipid–graphene hybrids. UV–Vis absorption spectra of: (**a**) alverine citrate (ALV) recorded in Milli-Q water and phosphate-buffered saline (PBS), showing characteristic absorption bands at ~215 nm and ~258 nm; (**b**) graphene oxide (GO) spectra before and after ultrasonication (usGO), illustrating increased absorbance intensity and improved dispersion after exfoliation; (**c**) PE-Rhod (1–20 µg/mL) vesicles at different concentrations, showing concentration-dependent changes in absorbance and a slight bathochromic shift associated with fluorophore interactions; (**d**) excitation (λem = 590 nm) and emission (λex = 561 nm) of SUV-Rhod with different amounts of usGO added.

The optical properties of alverine citrate (ALV), graphene oxide (GO), and rhodamine-labeled phospholipids (POPC-Rhod) were first analyzed to establish reference spectra for subsequent characterization of hybrid nanostructures. UV–Vis absorption spectra of the individual components are presented in Figure 1. ALV exhibited two characteristic absorption bands at approximately 215 nm (λ_1_) and 258 nm (λ_2_), attributed to electronic transitions associated with citrate carbonyl groups and aromatic π–π* transitions within the alverine moiety (Figure 1a) [30]. These spectral features were preserved in both ultrapure water and phosphate-buffered saline (PBS), indicating that moderate variations in ionic strength and pH did not significantly affect the electronic structure of the drug.

GO displayed a strong absorption peak centered at ~230 nm (λ_3_) with a shoulder near 300 nm (λ_4_), corresponding to π–π* transitions of sp^2^ carbon domains and n–π* transitions of carbonyl groups, respectively (Figure 1b) [20]. Ultrasonication resulted in a marked increase in absorbance intensity, consistent with improved exfoliation and dispersion of smaller GO nanosheets.

POPC-Rhod vesicles exhibited concentration-dependent changes in absorbance accompanied by a slight bathochromic shift (Figure 1c). The decrease in absorbance intensity with dilution followed Beer–Lambert behavior, whereas the red shift observed at higher concentrations suggests intermolecular interactions between rhodamine moieties embedded in the lipid bilayer.

The quantitative analysis of the absorbance vs. concentration dependence with the linear regression fitting plot is shown in Figure S1 (ESI).

#### 2.1.2. Formation and Optical Properties of POPC-Rhod@GO Hybrids

Hybrid lipid–graphene structures were obtained by incubating POPC-Rhod vesicles with ultrasonicated GO. UV–Vis spectra of the resulting dispersions showed the combined spectral features of both components, confirming successful hybrid formation (Figure S2). In particular, the characteristic GO absorption at ~230 nm was retained, while the rhodamine band in the visible range remained detectable but partially attenuated, indicating electronic interactions between the fluorophore and the graphene surface.

Fluorescence spectroscopy further supported hybrid formation. Progressive quenching of rhodamine emission was observed upon increasing GO concentration (Figure 1d), reaching a plateau at a lipid-to-GO mass ratio of approximately 0.76. This behavior suggests saturation of available adsorption sites on the graphene surface and is consistent with short-range energy or electron transfer processes between rhodamine molecules and GO nanosheets.

Extrusion of the dispersions for the formation of small unilamellar vesicles (SUVs) resulted in a reduction in the intensity of rhodamine absorption features, while the overall dispersion retained its characteristic color, indicating preservation of the fluorophore within the lipid bilayer. Difference spectra confirmed the continued presence of ALV in drug-loaded formulations after extrusion, even though the characteristic 215 nm peak became less pronounced.

### 2.2. Influence of Alverine on Lipid–Graphene Interactions

**Figure 2:**
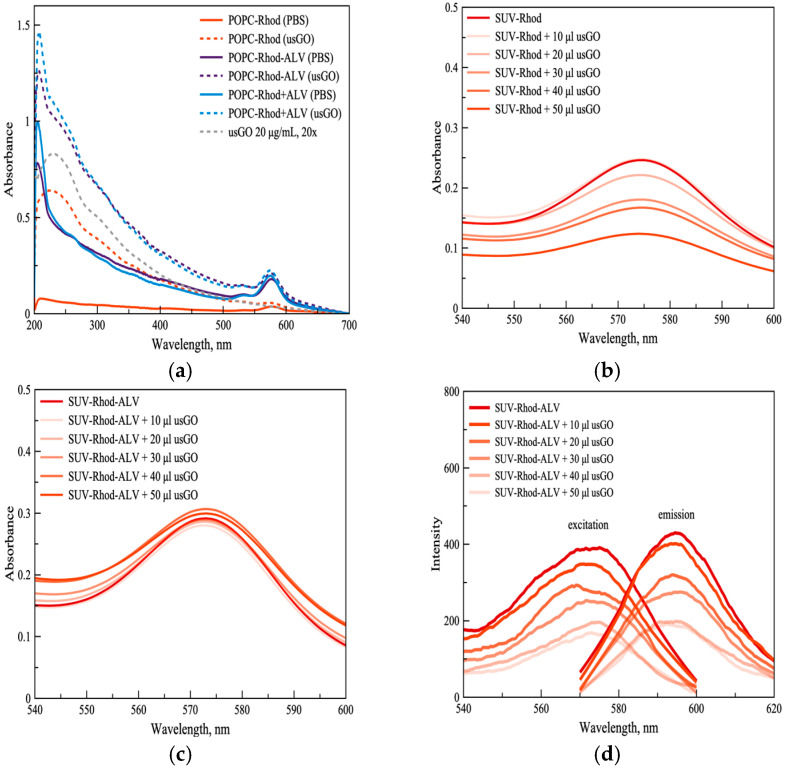
(**a**) UV–Vis absorption spectra of the lipid vesicle dispersions recorded prior to extrusion, measured for vesicles alone (solid lines) and in the presence of ultrasonicated graphene oxide (usGO, 20 μg mL^−1^, dashed lines). Spectra are shown for POPC-Rhod without drug and for alverine-loaded vesicles prepared by pre-loading (POPC-Rhod-ALV) and post-loading (POPC-Rhod+ALV) approaches. (**b**,**c**) UV–Vis spectra obtained during the titration experiment in which increasing amounts of usGO were added to SUV-Rhod (**b**) and SUV-Rhod-ALV (**c**) dispersions. (**d**) Fluorescence excitation (λem = 590 nm) and emission (λex = 561 nm) spectra of SUV-Rhod-ALV recorded at increasing usGO concentrations.

To investigate the effect of drug incorporation on hybrid formation, POPC-Rhod vesicles loaded with ALV using pre-loading (POPC-Rhod-ALV) and post-loading (POPC-Rhod+ALV) strategies were analyzed spectroscopically (Figure 2). UV–Vis spectra of the hybrid systems (Figure 2a) exhibited characteristic absorption peaks corresponding to GO (λ_3_ ≈ 230 nm; λ_4_ ≈ 300 nm) and ALV (λ_1_ ≈ 215 nm), confirming the presence of all components within the assemblies. Compared with drug-free POPC-Rhod@GO hybrids, ALV-containing systems showed enhanced absorbance in the UV region, suggesting additional electronic interactions within the composite structure.

The spectroscopic analysis of the POPC-Rhod before (multilamellar vesicles) and after (small unilamellar vesicles, SUVs) extrusion for the POPC-Rhod and ALV-loaded POPC-Rhod samples alone and corresponding hybrids with usGO was performed. All three samples showed that the Rhodamine characteristic absorption was less evident for the extruded samples; the dispersion still exhibited an intense color (Figure S2, ESI). Concerning the POPC-Rhod SUV, an increase and broadening of absorption is observed, alongside the loss of clear evidence of the peak and shoulder characteristic of the GO. As per the POPC-Rhod-ALV and POPC-Rhod+ALV SUVs, although both samples after the extrusion do not exhibit the peak at 215 nm anymore, the presence of ALV is still confirmed by the positive difference spectra compared to the GO alone.

As to the UV–Vis spectra recorded during titration with GO, a progressive decrease in absorbance was observed for SUV-Rhod (Figure 2b) but not for SUV-Rhod-ALV (Figure 2c). On the other hand, the most sensitive fluorescence spectra displayed a similar trend of decreased intensity by increased amount of usGO added (Figure 2d).

This effect can be explained by the fact that the process of energy transfer in a photosynthetic membrane typically takes place on a time scale from less than 100 fs to hundreds of ps. According to the different timescales of the photophysical process of absorption (typically in the order of 10^−15^ s) and fluorescence (from ns up to ms), the key role played by alverine in the process can be ruled out from the absorbance spectra but not from the emission spectra. In general, fluorescence measurements demonstrated that both loading strategies produced vesicles retaining rhodamine fluorescence; however, the presence of ALV slightly reduced the extent of fluorescence quenching induced by GO (see Figure 2d vs. Figure 1d). This observation indicates that drug molecules embedded in the lipid bilayer may influence the spatial arrangement of rhodamine groups relative to the graphene surface, thereby modulating fluorophore–graphene interactions. Despite these differences, both loading approaches yielded stable lipid–graphene hybrids, demonstrating that incorporation of ALV does not hinder lipid adsorption onto GO nanosheets.

### 2.3. Influence of ALV on Phospholipid Packing and Liposome Morphology

#### 2.3.1. Langmuir Monolayer Studies

**Figure 3:**
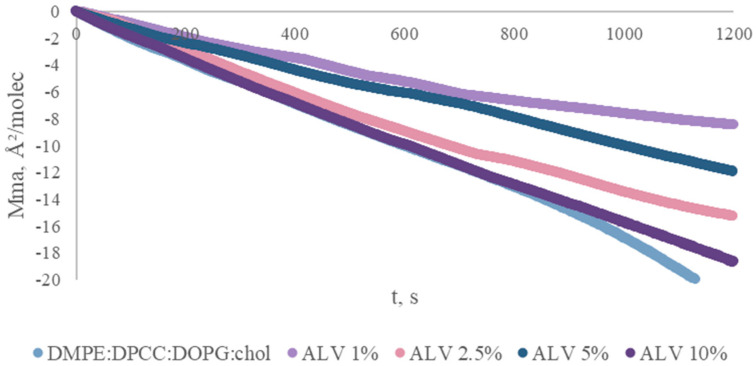
Relaxation kinetics of phospholipid monolayers containing different molar fractions of alverine citrate (0–10%) recorded at constant surface pressure. The decrease in mean molecular area over time indicates monolayer reorganization, while the reduced slope in the presence of alverine reflects stabilization of lipid packing.

Langmuir monolayer experiments were performed to evaluate the influence of ALV on phospholipid packing at the air–water interface. Relaxation curves recorded at constant surface pressure showed a gradual decrease in mean molecular area (Mma) over time, indicating ongoing monolayer reorganization (Figure 3).

The presence of ALV (1–10%) slowed the decrease in Mma, particularly at 1% and 5% molar fractions, suggesting stabilization of lipid packing.

Monolayers containing these ALV concentrations exhibited greater stability than those containing 2.5% and 10% drug, indicating a concentration-dependent effect of ALV on lipid organization. The findings from the lipid monolayer study suggest that the incorporation of alverine citrate into liposomes, which are composed of lipid bilayers, may also positively affect lipid vesicles, indicating concentration-dependent drug–lipid interactions.

#### 2.3.2. Size Distribution and Morphology of ALV-Loaded Liposomes

ALV-loaded liposomes were prepared by thin-film hydration followed by ultrasonication and characterized using dynamic light scattering (DLS) and atomic force microscopy (AFM). DLS measurements demonstrated that sonication significantly reduced vesicle size and polydispersity index (PDI), yielding small unilamellar vesicles with improved uniformity (Table 1, Figure S3). Liposomes containing 1% and 5% ALV displayed lower PDI values compared with those containing intermediate drug concentrations, suggesting that ALV influences bilayer packing and vesicle stability. This effect may be associated with the hydrophobic character of ALV, which promotes its incorporation into the lipid bilayer and may alter lipid packing density.

**Figure 4:**
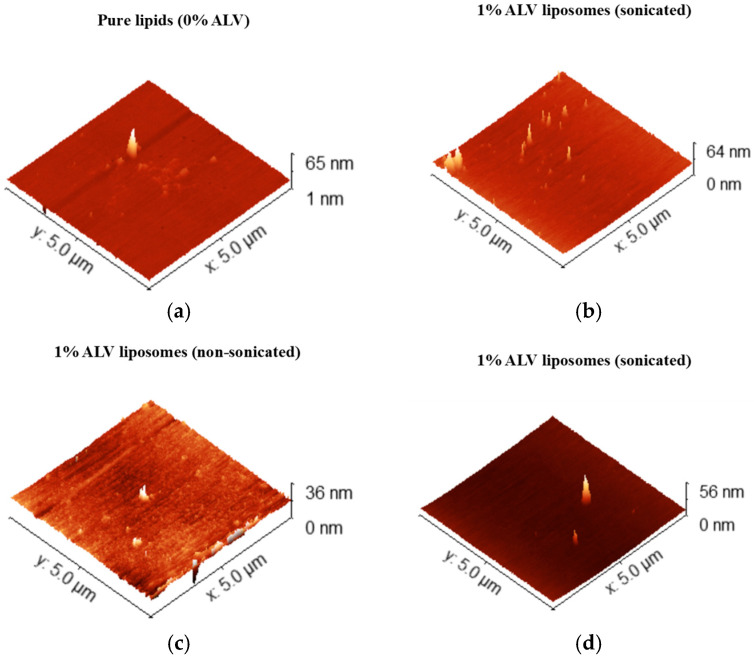
(**a**–**d**) Representative AFM topographical images of liposomes prepared with 0% (pure lipids, sonicated), 1% (sonicated), 1% (non-sonicated) and 5% (sonicated) ALV.

AFM topographical images confirmed the presence of nanoscale vesicular structures deposited on mica substrates (Figure 4).

Sonicated samples showed smaller and more uniformly distributed vesicles compared with non-sonicated preparations, which exhibited larger and heterogeneous structures indicative of multilamellar vesicles and aggregates.

Nanomechanical analysis revealed that sonicated liposomes exhibited reduced stiffness and Young’s modulus compared with non-sonicated counterparts, consistent with increased bilayer fluidity (Table 2).

Liposomes containing 1% ALV displayed the lowest Young’s modulus and highest deformation, indicating a softer and more elastic membrane, whereas formulations containing 5% ALV exhibited higher stiffness and lower adhesion energy, suggesting increased structural stability.

In detail, the analysis of AFM results indicated that the lowest height was exhibited by the non-sonicated ALV 1% sample, suggesting more compact surface features or reduced agglomeration compared to sonicated samples. However, the size of liposomes may be reduced with respect to their original diameter due to the method of sample preparation. The highest adhesion force was observed for the non-sonicated ALV 1% sample, indicating stronger tip–sample interactions. This may be due to higher surface compliance or more exposed adhesive components. In contrast, the sonicated ALV 5% sample showed the lowest adhesion force.

The lower adhesion force might be associated with the reduced ability of the formulation to bind with proteins when circulating in the bloodstream. On the other hand, it may also indicate a highly ordered and stable membrane. Also, the adhesion energy reached the highest value in the non-sonicated ALV 1% liposomes. This parameter represents the strength of physical interactions between the vesicle and other surfaces. The sonicated ALV 5% exhibited the lowest adhesion energy, indicating improved resistance to protein, which might be advantageous for transdermal drug delivery.

The lowest deformation occurred in the ALV 0% sample (10.3 nm), indicating higher resistance to indentation. The highest deformation was observed for the sonicated ALV 1% sample (36 nm), suggesting a softer or more elastic surface. This parameter gives more information about the possibility of penetrating the narrow pores in the stratum corneum. Energy dissipation increased with ALV content, with the highest values in the sonicated ALV 1% and 5% samples. This could reflect a more fluid or less stable bilayer mobility of the molecules. The highest Young’s modulus values were recorded for the non-sonicated ALV 1% and 5% samples, indicating enhanced stiffness. The sonicated ALV 1% sample showed the lowest modulus. A lower Young’s modulus indicates that the lipid bilayer may facilitate enhanced skin penetration.

The stiffness trend mirrors the modulus results, with the lowest value in the sonicated ALV 1% sample and the highest in the non-sonicated ALV 1%. Vesicle stiffness influences its release kinetics, cellular uptake and penetration efficiency [31].

### 2.4. Encapsulation Efficiency of Alverine

Encapsulation efficiency was evaluated using a dialysis-based method. During dialysis against PBS, no detectable ALV was observed in the external medium within the sensitivity limits of UV–Vis analysis, indicating minimal drug leakage and suggesting efficient retention of ALV within the lipid bilayer. Although quantitative loading values could not be precisely determined due to detection limitations, these results support stable incorporation of ALV in the liposomal formulations.

### 2.5. Cell-Free Experiments

**Figure 5:**
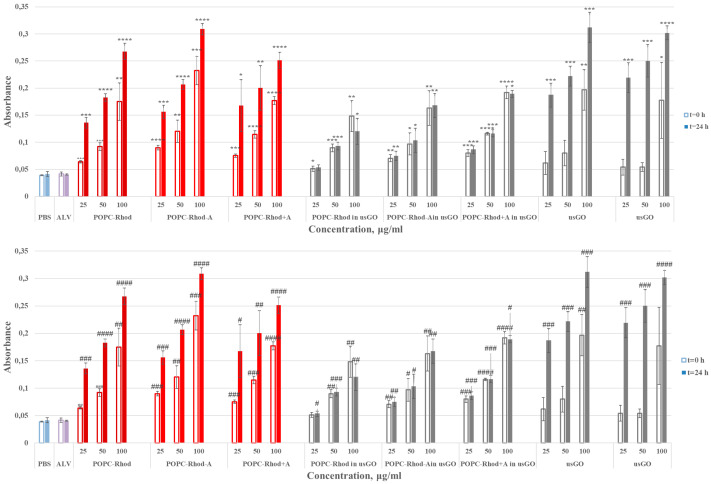
Absorbance values for POPC-Rhod, POPC-Rhod-ALV (POPC-Rhod-A), POPC-Rhod+ALV (POPC-Rhod+A), and POPC-Rhod, POPC-Rhod-A, POPC-Rhod+A prepared in usGO dispersion. The PBS (**top**) and ALV (**bottom**) samples were used as a negative and positive control (group 2), respectively. The hollow bars correspond to the experiment at t = 0 h, and the filled bars correspond to the experiment at t = 24 h, as described above. Data are expressed as mean ± S.E.M. (n = 3). Statistical significance was determined relative to untreated control samples (* *p* < 0.05, ** *p* < 0.01, *** *p* < 0.001, **** *p* < 0.0001) or ALV-treated samples (^#^ *p* < 0.05, ^##^ *p* < 0.01, ^###^ *p* < 0.001, ^####^ *p* < 0.0001).

**Figure 6:**
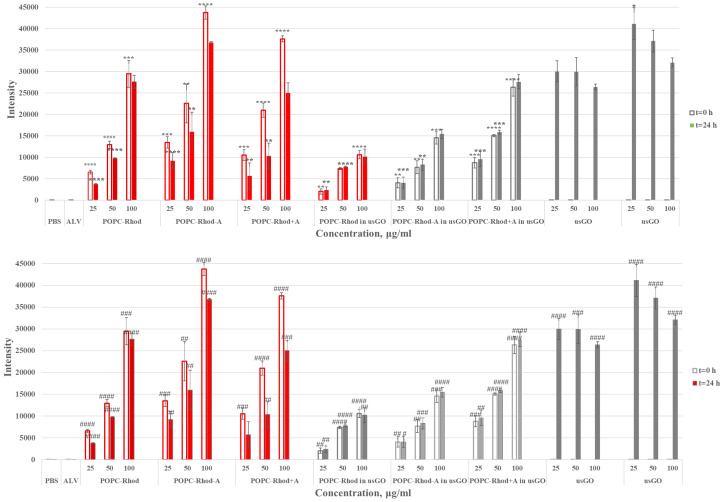
Intensity values for POPC-Rhod, POPC-Rhod-ALV (POPC-Rhod-A), POPC-Rhod+ALV (POPC-Rhod+A), and POPC-Rhod, POPC-Rhod-A, POPC-Rhod+A prepared in usGO dispersion. The PBS (**top**) and ALV (**bottom**) samples were used as a negative and positive control (group 2), respectively. The hollow bars correspond to the experiment at t = 0 h, and the filled bars correspond to the experiment at t = 24 h, as described above. Data are expressed as mean ± S.E.M. (n = 3). (* *p* ≤ 0.05, ** *p* ≤ 0.01, *** *p* ≤ 0.001, **** *p* ≤ 0.0001 vs. CTRL; ^#^ *p* ≤ 0.05, ^##^ *p* ≤ 0.01, ^###^ *p* ≤ 0.001, ^####^ *p* ≤ 0.0001 vs. ALV).

Cell-free spectroscopic measurements were carried out to scrutinize the stability and optical properties of the ALV-loaded POPC-Rhod@GO nanoplatforms (Figure 5 and Figure 6).

Most samples exhibit a linear increase in absorbance and emission values with increasing sample concentration, consistent with stable dispersions and the absence of significant aggregation. Addition of GO to POPC-Rhod-containing samples resulted in an increase in overall absorbance and fluorescence signals, while incorporation of lipid vesicles into GO dispersions produced a marked enhancement of optical responses compared with GO alone. These results indicate strong interactions between lipid vesicles and graphene nanosheets and confirm the formation of stable hybrid nanostructures in aqueous media.

### 2.6. Cellular Uptake of Hybrid Nanostructures

**Figure 7:**
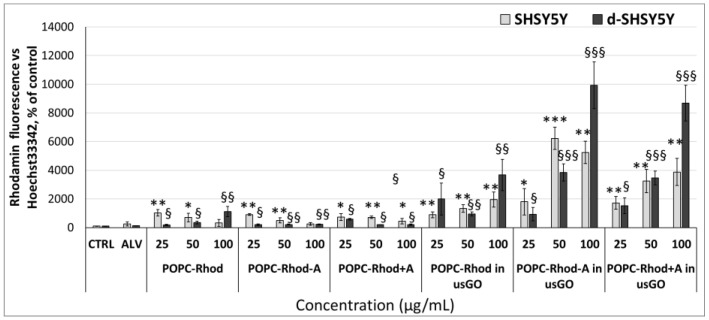
Quantitative analysis of cellular uptake of rhodamine-labeled formulations in undifferentiated SH-SY5Y and differentiated d-SH-SY5Y cells after 24 h incubation. Fluorescence intensity was normalized to Hoechst nuclear staining and expressed as a percentage relative to untreated controls. Data are presented as mean ± SD (n = 3). Statistical comparisons were performed using one-way ANOVA with Tukey’s post hoc test (* *p* ≤ 0.05, ** *p* ≤ 0.01, *** *p* ≤ 0.001, vs. SH-SY5Y control untreated cells; § *p* ≤ 0.05, §§ *p* ≤ 0.01, §§§ *p* ≤ 0.001, vs. d-SH-SY5Y control untreated cells).

The internalization of rhodamine-labeled formulations was quantified in SH-SY5Y neuroblastoma cells and their differentiated neuronal-like variant (d-SH-SY5Y) [32,33] after 24 h incubation (Figure 7).

In undifferentiated cells, free POPC-Rhod vesicles exhibited moderate uptake (reaching 980 ± 210% at 25 µg/mL, 720 ± 190% at 50 µg/mL and 360 ± 140% at 100 µg/mL compared with the control, *p* < 0.05 for 25 µg/mL), while incorporation of GO significantly enhanced internalization in a concentration-dependent manner. The highest fluorescence intensities were observed for POPC-Rhod-ALV@GO hybrids (1800 ± 900% at 25 µg/mL, 6200 ± 750% at 50 µg/mL and 5200 ± 800% at 100 µg/mL, *p* < 0.001), indicating synergistic effects of lipid and graphene components on cellular uptake. The POPC-Rhod+ALV formulation in usGO demonstrated a comparable but less pronounced effect (1700 ± 420%, 3200 ± 850%, 3900 ± 950% respectively; *p* < 0.01–0.001).

In contrast, ALV alone did not significantly affect the uptake of lipid vesicles (POPC-Rhod-ALV: 520 ± 160%, 310 ± 120%, 210 ± 90% for 25 µg/mL, 50 µg/mL and 100 µg/mL, respectively; *p* > 0.05), consistent with the literature, which suggests that ALV does not possess intrinsic endocytosis-enhancing properties and does not affect membrane permeability in its free form [34].

Differentiated d-SH-SY5Y cells displayed generally higher levels of nanoparticle internalization than undifferentiated cells, which is consistent with data on increased endocytic activity and a more organized membrane architecture of neuron-like cells [33]. GO-containing formulations again showed the most pronounced uptake, with POPC-Rhod-ALV@GO hybrids producing the strongest intracellular fluorescence signals. In particular, free POPC-Rhod reached 1150 ± 300% at 100 µg/mL (*p* < 0.05), whereas its combinations with ALV without usGO did not differ from the lipid component (*p* > 0.05). POPC-Rhod in usGO caused an increase in fluorescence of up to 2000 ± 1100% at 25 µg/mL, 980 ± 220% at 50 µg/mL and 3700 ± 1100% at 100 µg/mL (*p* < 0.01–0.001), while for POPC-Rhod-ALV in usGO, the maximum values reached 3800 ± 600% at 25 µg/mL, 9900 ± 1600% at 50 µg/mL and 8700 ± 1200% at 100 µg/mL (*p* < 0.001). This effect is consistent with the literature, which indicates that hybrid lipid–graphene systems provide more efficient intracellular delivery due to the combination of the membrane compatibility of lipids and the high adsorption capacity of graphene [35,36,37]. These results demonstrate that GO plays a key role in promoting cellular internalization of lipid nanocarriers, while ALV does not independently influence uptake efficiency.

### 2.7. Cytotoxicity, Oxidative Stress, and Cellular Response

**Figure 8:**
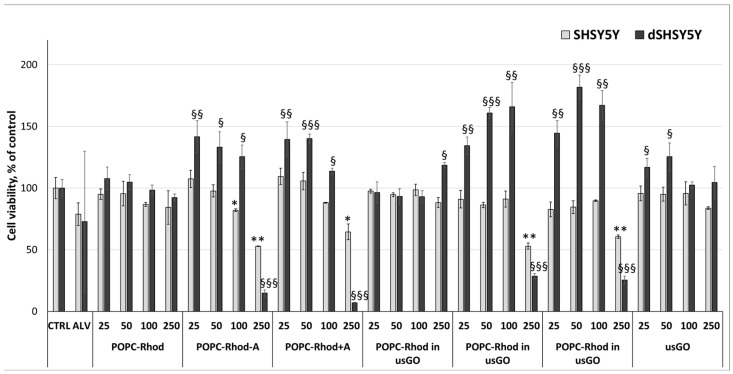
The results of MTT assay (±SD) for SH-SY5Y neuroblastoma cells, undifferentiated and differentiated, treated with POPC-Rhod, POPC-Rhod-ALV, POPC-Rhod+ALV, and POPC-Rhod, POPC-Rhod-ALV, POPC-Rhod+ALV prepared in usGO dispersion, as well as in PBS, ALV and usGO alone. The PBS was used as a negative control (* *p* ≤ 0.05, ** *p* ≤ 0.01, vs. SH-SY5Y control untreated cells; § *p* ≤ 0.05, §§ *p* ≤ 0.01, §§§ *p* ≤ 0.001, vs. d-SH-SY5Y control untreated cells).

**Figure 9:**
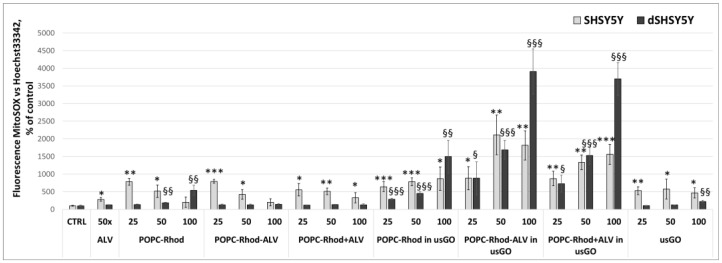
The results of MitoSOX mitochondrial ROS assay (±SD) for SH-SY5Y neuroblastoma cells, undifferentiated and differentiated, treated with POPC-Rhod, POPC-Rhod-ALV, POPC-Rhod+ALV, and POPC-Rhod, POPC-Rhod-ALV, POPC-Rhod+ALV prepared in usGO dispersion, as well as in PBS, ALV and usGO alone. The PBS was used as a negative control (* *p* ≤ 0.05, ** *p* ≤ 0.01, *** *p* ≤ 0.001, vs. SH-SY5Y control untreated cells; § *p* ≤ 0.05, §§ *p* ≤ 0.01, §§§ *p* ≤ 0.001, vs. d-SH-SY5Y control untreated cells).

MTT and MitoSOX assays were used to evaluate cell viability and mitochondrial reactive oxygen species (ROS) production following exposure to the different formulations (Figure 8 and Figure 9).

While the MTT assay aims to evaluate cell viability based on mitochondrial metabolic activity, the MitoSOX assay assesses mitochondrial superoxide production, indicating oxidative stress.

The results of MTT (Figure 8) showed that in undifferentiated SH-SY5Y cells, most formulations exhibited concentration-dependent cytotoxicity, particularly at the highest tested concentration (250 µg mL^−1^). The most pronounced toxic effect was observed for the formulations POPC-Rhod-ALV, POPC-Rhod+ALV (18 ± 1% and 12 ± 1% for 50 µg/mL, respectively), as well as their combinations with GO. At the maximum concentration (250 µg/mL), the reduction in cell viability reached approximately 50% compared with the control, indicating a significant increase in cell death.

In contrast, differentiated cells showed greater resistance to treatment, and moderate concentrations of ALV-containing formulations were associated with maintained or slightly increased viability. The POPC-Rhod-ALV and POPC-Rhod+ALV formulations not only had no cytotoxic effect but also contributed to an increase in cell viability in the concentration range of 25–100 µg/mL. However, as concentrations increased from 25 to 100 µg/mL, a moderate reduction in this effect was noted; however, in any case, the viability level remains higher than or equivalent to the control.

Concerning the MitoSOX assay, Figure 9 shows that in undifferentiated SH-SY5Y cells, there is a slight increase in mitochondrial ROS levels in the presence of POPC-Rhod with and without ALV at the lowest concentrations, which may indicate the initial cellular response to interaction with lipid nanostructures. However, upon increasing the concentration to 50 and 100 μg/mL, ROS levels decreased to values comparable to the control, indicating the absence of a sustained pro-oxidant effect of these compounds. In differentiated d-SH-SY5Y cells, POPC-Rhod, both in the presence and absence of ALV, did not induce an increase in ROS production at any of the tested concentrations, with values remaining at control levels.

The effect of the usGO carrier should be noted specifically. In undifferentiated SH-SY5Y cells, its presence led to an increase in mitochondrial ROS levels at all tested concentrations, which is in line with the previously observed slight increase in cytotoxicity and may be associated with the induction of oxidative stress. At the same time, no similar effect was observed in differentiated d-SH-SY5Y cells, confirming their cytostability to graphene nanostructures.

A different pattern was observed when combined POPC-Rhod formulations with ALV were used in the presence of usGO. In this case, a marked concentration-dependent increase in the level of mitochondrial ROS was observed in both SH-SY5Y and d-SH-SY5Y. The most significant effect was recorded at a concentration of 100 μg/mL for the POPC-Rhod-ALV and POPC-Rhod+ALV formulations, particularly in differentiated cells. These results indicate that the combination of a lipid component, ALV and a graphene carrier may lead to an increase in mitochondrial oxidative stress, despite the previously observed neuroprotective effect in the range of moderate concentrations.

Despite the elevated oxidative stress indicated by MitoSOX, the MTT assay confirmed that the cells remained metabolically active and viable. This suggests a possible adaptive response; the cells experience oxidative stress but have not yet undergone apoptosis. The large discrepancy between MitoSOX and MTT results may also stem from differences in assay duration and sensitivity: MitoSOX detects early oxidative events, whereas MTT reflects overall cellular metabolic viability. GO alone did not significantly affect the viability of differentiated cells but induced a moderate increase in mitochondrial ROS in undifferentiated cells. Hybrid formulations containing both ALV and GO produced a concentration-dependent increase in ROS levels, especially at higher doses, indicating that combined lipid–drug–graphene systems may enhance oxidative stress under certain conditions.

**Figure 10:**
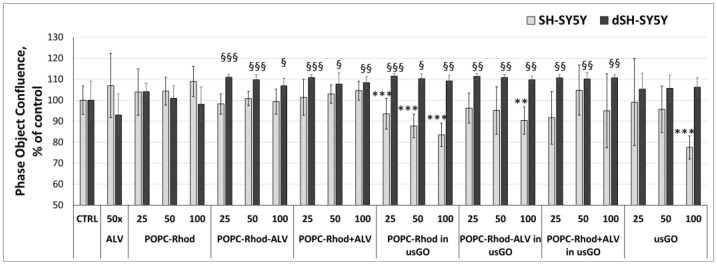
Average values (±S.D.) of real-time cytotoxicity and proliferation assessed over 24 h using the IncuCyte^®^ Live-Cell Analysis System in undifferentiated SH-SY5Y and differentiated d-SH-SY5Y cells. Cells were treated with POPC-Rhod, POPC-Rhod-ALV, POPC-Rhod+ALV, and POPC-Rhod, POPC-Rhod-ALV, POPC-Rhod+ALV prepared in usGO dispersion, as well as PBS, ALV and usGO alone. The untreated sample, i.e., cells in medium, was used as a negative control (** *p* ≤ 0.01, *** *p* ≤ 0.001, vs. SH-SY5Y control untreated cells; § *p* ≤ 0.05, §§ *p* ≤ 0.01, §§§ *p* ≤ 0.001, vs. d-SH-SY5Y control untreated cells).

Figure 10 shows the results of cytotoxicity/proliferation on cancer (SH-SY5Y) and differentiated (d-SHSY5Y) neuroblastoma cells.

Overall, the tested treatments exerted a more pronounced effect on differentiated d-SH-SY5Y cells, indicating a differentiation-dependent response and suggesting a potential neuroprotective profile under these conditions. In undifferentiated SH-SY5Y cells, POPC-Rhod formulated in usGO significantly decreased cell viability at all tested concentrations. A comparable reduction in viability was observed for the highest concentration of POPC-Rhod-ALV and for usGO alone, indicating a concentration-dependent cytotoxic effect in proliferating neuroblastoma cells. In contrast, differentiated d-SH-SY5Y cells were less susceptible to cytotoxicity. ALV alone demonstrated a mild antiproliferative effect in these cells. Importantly, POPC-Rhod-ALV and POPC-Rhod+ALV, both in the presence and absence of usGO, as well as POPC-Rhod formulated in usGO, exhibited a statistically significant neuroprotective effect in differentiated cells compared with the untreated control. These findings suggest that the combined formulations may selectively reduce viability in undifferentiated neuroblastoma cells while preserving or improving survival in differentiated neuronal-like cells.

### 2.8. Confocal Microscopy Analysis of Intracellular Localization

**Figure 11:**
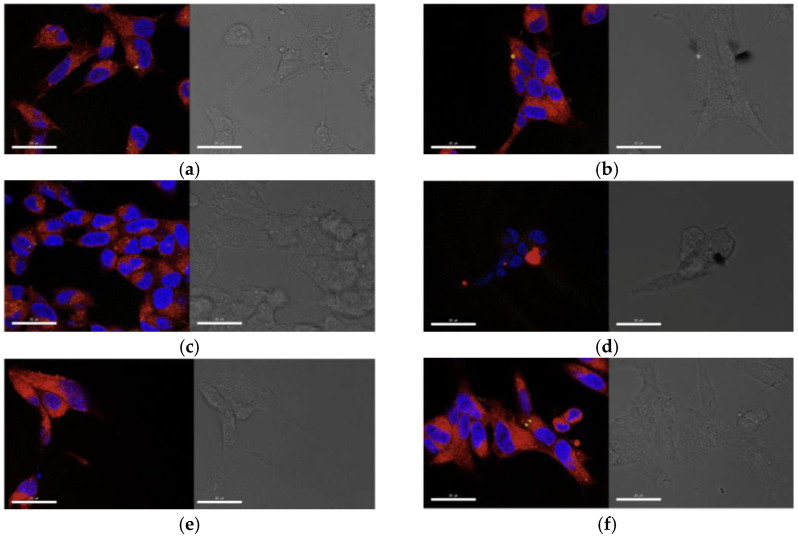
LSM representative micrographs of retinoic acid-differentiated neuroblastoma cells. Cells were untreated (**a**) or treated with ALV (**b**), POPC-Rhod (**c**), usGO alone (**d**), POPC-Rhod-ALV (**e**) and POPC-Rhod+ALV (**f**). For each condition, the left panel shows merged fluorescence images of nuclei (blue, Hoechst), lysosomes (green, Lysotracker Green) and mitochondria or Rhod-labeled formulations (red, Mitotracker Deep Red/Rhodamine). The right panel displays the corresponding bright-field (BF) micrographs. Scale bar = 30 μm.

Confocal laser scanning microscopy confirmed efficient internalization of rhodamine-labeled formulations in differentiated SH-SY5Y cells (Figure 11). POPC-Rhod and ALV-loaded liposomes produced strong intracellular fluorescence signals with predominantly cytoplasmic localization. Cells treated with GO alone displayed sheet-like structures consistent with internalized graphene nanosheets but retained overall cellular morphology.

Hybrid POPC-Rhod-ALV@GO formulations exhibited punctate intracellular fluorescence patterns, suggesting vesicular uptake and accumulation in intracellular compartments. Importantly, no major morphological alterations or nuclear damage were observed in differentiated cells, supporting the overall biocompatibility of the nanoplatforms at the tested concentrations. As shown in Figure 11, LSM imaging revealed clear morphology- and treatment-dependent differences in retinoic acid–differentiated neuroblastoma cells. Untreated cells (a) displayed typical neuronal-like morphology with elongated processes and well-preserved mitochondrial networks (red) distributed throughout the cytoplasm, while lysosomes (green) exhibited a perinuclear localization pattern. ALV-treated cells (b) maintained overall cellular architecture comparable to the control, with no evident disruption of mitochondrial organization or lysosomal distribution. Treatment with POPC-Rhod (c) resulted in a pronounced intracellular red fluorescence signal, confirming efficient cellular uptake of the Rhod-labeled formulation. The fluorescence appeared mainly cytoplasmic, partially overlapping with mitochondrial staining, while cell morphology remained largely preserved. Cells exposed to usGO alone (d) did not exhibit marked alterations in nuclear morphology or overall cellular structure, although intracellular or membrane-associated dark, sheet-like structures consistent with 2D nanosheet infiltration were clearly evident in the bright-field images. In contrast, cells treated with POPC-Rhod-ALV (e) showed a more punctate and localized red fluorescence pattern, suggesting intracellular accumulation of the conjugated formulation, accompanied by slight morphological alterations in some cells. Notably, POPC-Rhod+ALV-treated cells (f) displayed strong intracellular fluorescence with preserved neuronal-like morphology and intact nuclei, indicating effective uptake without evident structural damage. Bright-field images corroborated the fluorescence observations, confirming that differentiated cells largely retained their characteristic morphology across treatments. Collectively, these data support efficient internalization of the tested formulations in differentiated neuroblastoma cells, with no evident severe morphological impairment, consistent with the neuroprotective profile observed in the viability assays.

## 3. Discussion

The present study demonstrates the feasibility of constructing graphene oxide-supported lipid bilayer hybrids capable of delivering a repurposed small-molecule drug while enabling fluorescence-based cellular tracking [27,38].

Alverine-loaded, rhodamine-labeled POPC liposomes were successfully integrated with graphene oxide nanosheets to generate a multifunctional nanoplatform combining drug delivery, imaging capability, and graphene-mediated biointerface activity.

Spectroscopic characterization confirmed strong interactions between the lipid, fluorophore, and GO components, manifested by fluorescence quenching and changes in absorbance profiles. The progressive decrease in rhodamine emission upon addition of ultrasonicated GO is characteristic of efficient electronic coupling between the fluorophore and the graphene surface, consistent with energy or electron transfer processes at the lipid–graphene interface. Such quenching phenomena are widely reported in graphene–fluorophore systems and indicate close spatial proximity between the lipid bilayer and the GO surface [27,38].

Complementary physicochemical characterization using dynamic light scattering and atomic force microscopy revealed that sonication produced small unilamellar vesicles with reduced polydispersity, increased deformability, and decreased stiffness. These features are generally associated with improved membrane interaction and cellular uptake, suggesting that the processing conditions employed here generated liposomes with properties favorable for biological applications. Langmuir monolayer experiments further indicated that alverine interacts with phospholipid membranes in a concentration-dependent manner, stabilizing lipid packing at intermediate molar fractions. This behavior is consistent with the hydrophobic nature of alverine [39] and supports its preferential incorporation within the lipid bilayer rather than adsorption onto graphene surfaces.

Confocal microscopy and quantitative fluorescence analysis confirmed efficient internalization of POPC-Rhod@GO hybrids in both undifferentiated and differentiated SH-SY5Y cells, with particularly strong intracellular fluorescence observed for graphene-containing formulations. The presence of GO nanosheets likely enhances membrane adhesion and facilitates endocytic uptake, consistent with previous reports describing graphene-induced modulation of membrane curvature and endocytosis [40].

In both cell phenotypes, the POPC-Rhod-ALV formulation in usGO consistently demonstrated the highest level of intracellular fluorescence, indicating the greatest cellular penetration. These findings support the role of GO as an active structural scaffold that increases nanoparticle–cell interactions and promotes internalization of lipid-based carriers.

A pronounced dependence of cellular response on the differentiation state of SH-SY5Y cells was observed, highlighting the importance of cellular phenotype in nanomedicine evaluation. Undifferentiated neuroblastoma cells exhibited higher susceptibility to graphene-containing formulations, with significant reductions in metabolic viability at elevated concentrations. Such cytotoxic and stress-mediated effects of GO nanomaterials have been documented in cancer cells and are thought to involve ROS induction and mitochondrial impairment [41].

In contrast, differentiated neuronal-like cells exhibited greater tolerance to the same treatments, maintaining high viability across moderate concentration ranges.

This divergence likely reflects differences in membrane composition, metabolic activity, and antioxidant defenses between proliferating and differentiated cells [42]. Rapidly dividing cells are generally more sensitive to oxidative stress and membrane perturbation, which may explain the enhanced cytotoxicity of GO-containing formulations in undifferentiated SH-SY5Y cells.

MitoSOX and MTT assays revealed a complex interplay between mitochondrial reactive oxidative stress generation and cell viability. Elevated mitochondrial superoxide levels were observed particularly in treatments combining POPC-Rhod with usGO, indicating that GO may promote ROS generation, as previously reported for graphene-based nanomaterials [43,44]. However, the absence of immediate viability loss at moderate concentrations suggests that cells mount adaptive responses to moderate oxidative stress, highlighting the necessity of assessing both early and late markers of cell fate in nanotoxicity evaluations.

The apparent discrepancies between mitochondrial ROS measurements and metabolic viability assays can be attributed to the different biological processes detected by these methods. MitoSOX staining reports early mitochondrial superoxide production, whereas MTT reduction reflects overall cellular metabolic activity. The observation of elevated ROS levels without immediate loss of viability suggests that oxidative stress precedes detectable metabolic impairment and may only translate into cytotoxicity upon prolonged exposure or at higher doses.

Notably, in undifferentiated SH-SY5Y cells, increased ROS levels correlated with reduced viability, whereas in differentiated cells, a moderate rise in ROS was not accompanied by significant cytotoxicity. This phenotype-dependent response indicates that neuronal-like cells possess more effective mechanisms to buffer oxidative stress. Alverine alone exhibited a mild antiproliferative effect in neuroblastoma cells, aligning with reports suggesting that the drug may modulate signaling pathways associated with cell survival and growth. Incorporation of alverine into lipid–graphene hybrids did not markedly alter cellular uptake but appeared to influence downstream cellular responses, suggesting that the drug contributes to the biological activity of the nanoplatform beyond passive delivery. The overall therapeutic profile of the hybrid systems likely arises from the combined effects of enhanced intracellular delivery, GO-induced modulation of cellular redox status, and alverine-mediated signaling effects.

Although the system was conceived as a theranostic platform, in the present study, the diagnostic functionality relies on rhodamine fluorescence as a model imaging modality. The developed nanostructure should therefore be considered a fluorescence-traceable therapeutic carrier rather than a clinically optimized theranostic system. Nevertheless, the modular architecture of the lipid–graphene hybrid allows straightforward substitution of the fluorophore with clinically relevant imaging agents or the co-loading of additional therapeutic drugs, providing a flexible foundation for future multifunctional nanomedicine development.

We also note that GO-based hybrids have previously been explored for anti-angiogenic and anti-proliferative effects in tumor models [43,45], further supporting the rationale for their application in targeted cancer therapy. These studies collectively reinforce the dual utility of GO: as a structural platform for multifunctional nanomedicine and as an active modulator of cellular behavior.

Encapsulation efficiency could not be quantified precisely because alverine concentrations in the dialysate remained below the detection limit. Furthermore, drug release kinetics, long-term colloidal stability, and in vivo biodistribution were not evaluated. All biological experiments were conducted in two-dimensional in vitro models, which cannot fully reproduce the complexity of tumor microenvironments or systemic exposure conditions.

Despite these limitations, the results highlight the potential of graphene oxide–lipid hybrids as adaptable nanocarriers for the delivery of repurposed drugs to neural cancer cells. The observed phenotype-dependent cytotoxicity, combined with efficient fluorescence-based tracking, supports further investigation of this platform in advanced three-dimensional cell models and in vivo systems.

## 4. Materials and Methods

### 4.1. Chemicals and Physicochemical Characterization of ALV-Loaded POPC-Rhod@GO Hybrids

Alverine citrate powder was kindly provided by Synteza sp. z o.o.. (Poznań, Poland). Phosphate-buffered saline (PBS, pH 7.4; 137 mM NaCl, 2.7 mM KCl, 10 mM phosphate, Amresco, Inc., Solon, OH, USA, supplied by VWR International, Europe) and ultrapure Milli-Q water (18.2 MΩ·cm) were used throughout the study. Graphene oxide (GO, 0.4% *w*/*v* aqueous dispersion) was obtained from Graphenea Inc. (San Sebastián, Spain). 1-palmitoyl-2-oleoyl-sn-glycero-3-phosphocholine (POPC), and rhodamine-labeled phosphoethanolamine (PE-Rhod) were purchased from Avanti Polar Lipids (Alabaster, AL, USA).

UV–Vis spectra were recorded on a UV–Vis spectrophotometer Lambda 365 (PerkinElmer, Inc., Shelton, CT, USA) in quartz cuvettes with a path length of 1 cm. Spectra were processed using MagicPlot 3.0.1 software.

The GO stock dispersion was diluted in PBS to 4 mg/mL and ultrasonicated for 2 h using an UP50H ultrasonic processor (Hielscher Ultrasonics GmbH, Teltow, Germany) at 100% amplitude and 1 cycle in an ice bath to prevent thermal degradation. For spectroscopic measurements, ultrasonicated GO (usGO) was further diluted to 20 μg/mL.

To determine the absorption maximum of PE-Rhod, spectra were collected at multiple dilutions and first-derivative analysis was performed using Origin 2025 (OriginLab, Northampton, MA, USA). The wavelength corresponding to zero derivative was taken as the absorption maximum (λmax = 574 nm) (Table S1, Figures S4 and S5 in ESI).

### 4.2. Small Unilamellar Vesicles (SUVs) Preparation and ALV Loading

SUVs were prepared using the thin film hydration method followed by membrane extrusion [28]. POPC and PE-Rhod were dissolved in chloroform and transferred into a round-bottom flask. The solvent was evaporated under a nitrogen stream, and residual solvent was removed under vacuum to form a thin lipid film.

The film was hydrated with PBS (pH 7.4) to obtain a final POPC concentration of 5 mg/mL. For pre-loading experiments, alverine citrate was included in the lipid mixture prior to solvent evaporation. For post-loading experiments, alverine was added during hydration. The suspension was extruded through polycarbonate membranes (100 nm followed by 30 nm pore size) using a manual extruder (Hamilton syringes) for 11–13 passes to obtain monodisperse SUVs. Samples were stored under nitrogen at 5 °C and protected from light.

SUV dispersions were diluted to a final POPC concentration of 100 μg/mL in PBS or in usGO (20 μg/mL) prior to spectroscopic measurements.

The list of samples with exact amounts of reagents is presented in Table S2 (ESI).

### 4.3. Spectroscopic and Fluorimetric Analysis

Titration experiments were performed by incremental addition of usGO to SUV-Rhod and SUV-Rhod-ALV dispersions. UV–Vis spectra were recorded using a Lambda 365 spectrophotometer, and fluorescence spectra were measured using an LS 55 spectrofluorimeter (PerkinElmer, Inc., Shelton, CT, USA). Fluorescence excitation and emission spectra were recorded at λex = 561 nm and λem = 600 nm. Time-dependent measurements were conducted after 24 h of incubation.

High-throughput fluorescence and absorbance measurements were performed using a Synergy H1 multimode microplate reader (BioTek Instruments, Inc., Winooski, VT, USA). Absorbance was recorded at 570 nm. Data were collected in triplicate and expressed as mean ± standard deviation. Statistical significance was evaluated using a two-tailed Student’s *t*-test.

### 4.4. Langmuir Monolayer Experiments

Lipid monolayers composed of cholesterol, DMPE, DPPC, and DOPG were spread on ultrapure water in a Langmuir trough (KSV NIMA, KSV Instruments, Ltd., Helsinki, Finland). Alverine citrate was added at 1–10 mol%. Surface pressure–area isotherms and relaxation curves were recorded to evaluate drug–lipid interactions.

### 4.5. Atomic Force Microscopy

Nanomechanical properties of liposomes were characterized using an NX10 atomic force microscope (Park Systems, Suwon, South Korea) in PinPoint mode. Liposomes were immobilized on poly-L-lysine-coated glass and imaged in liquid. Parameters such as height, deformation, adhesion force, and Young’s modulus were analyzed using Gwyddion software (Version 2.63, Nečas & Klapetek, Brno, Czech Republic).

### 4.6. Dynamic Light Scattering

Hydrodynamic diameter and polydispersity index of liposomes were measured using a Malvern Zetasizer (Malvern Panalytical, Malvern, Worcestershire, UK) at 37 °C.

### 4.7. Encapsulation Efficiency by Dialysis

Liposome dispersions were placed in dialysis tubing and immersed in PBS at 37 °C under stirring. Samples were collected from the external medium after 24 h and analyzed by HPLC to estimate the fraction of non-encapsulated alverine.

### 4.8. In Vitro Cellular Studies

#### 4.8.1. Chemicals for In Vitro Experiments

Dulbecco’s modified Eagle medium/Ham’s F-12 medium (DMEM/F12), Dulbecco’s modified Eagle medium (DMEM) high glucose, fetal bovine serum (FBS), all-trans-retinoic acid (RA), 3-(4,5-dimethylthiazol-2-yl)−2,5-diphenyltetrazolium bromide (MTT), and DMSO were provided by Sigma-Aldrich (St. Louis, MO, USA). Streptomycin and L-glutamine were provided by Lonza Group (Verviers, Belgium).

#### 4.8.2. Cell Culture and Differentiation

Human neuroblastoma SH-SY5Y cells (ATCC, Manassas, VA, USA) were cultured in complete cell culture medium, i.e., DMEM/F12, supplemented with 10% FBS, 2 mM L-glutamine and 100 U/mL of penicillin/streptomycin, at 37 °C under 5% CO_2_ atmosphere (Heraeus Hera Cell 150C incubator, Heraeus Instruments GmbH, Hanau, Germany). For neuronal differentiation, cells were seeded at 50–60% confluence in complete DMEM/F12 medium supplemented with 10% FBS and, after 24 h, the medium was replaced with DMEM high glucose containing 1% FBS and 10 μM all-trans retinoic acid. Differentiation medium (0.5% FBS, 10 μM retinoic acid) was refreshed every 48 h for 7 days (Figure S6, ESI).

#### 4.8.3. Cellular Uptake Studies

Cells were seeded at a density of 7 × 10^4^ cells/mL in 96-well plates (Corning^®^ tissue-culture treated culture 96-multiwell, Sigma-Aldrich, St. Louis, MO, USA) in full medium and incubated with fluorescent nanoformulations for 24 h. After treatment, cells were washed with PBS and intracellular fluorescence was measured using a Varioskan Flash multimode reader (Thermo Fisher Scientific, Vantaa, Finland).

Rhodamine fluorescence was recorded at λex = 553 nm and λem = 577 nm. Hoechst 33342 staining (λex = 361 nm, λem = 497 nm) was used to normalize fluorescence intensity to cell number.

#### 4.8.4. Cytotoxicity Assessment (MTT Assay)

Cell viability was evaluated using the standard MTT assay, as previously described [46]. SH-SY5Y and differentiated cells were seeded in 96-well plates and exposed to nanoformulations (25–250 μg/mL) for 48 h. After treatment, MTT was added to a final concentration of 0.5 mg/mL and incubated for 90 min. The supernatant was removed and the purple formazan crystals were resuspended in 100 μL of DMSO until complete dissolution. Absorbance was measured at 570 nm using the Varioskan^®^ Flash spectral scanning multimode reader (Waltham, MA, USA). Experiments were performed at least in triplicate. Results were expressed as % of viable cells vs. the concentration of each sample and expressed as mean ± SD.

#### 4.8.5. Mitochondrial Reactive Oxygen Species Measurement

Mitochondrial superoxide production was assessed using the MitoSOX probe. Cells were incubated with test formulations for 48 h, washed, and stained with 5 μM MitoSOX in PBS for 30 min at 37 °C. Fluorescence was measured by Varioskan^®^ Flash spectral scanning multimode reader at λex = 510 nm and λem = 580 nm and normalized to Hoechst 33342 (ex = 361 nm, em = 497 nm) fluorescence. Data are presented as the means ± SD of three replicas.

#### 4.8.6. Live-Cell Imaging and Proliferation Analysis

Cell proliferation and morphology were monitored in real time using the IncuCyte^®^ Live-Cell Analysis System (Sartorius AG, Göttingen, Germany). Images were acquired every hour for 24 h and cell confluence was quantified using the IncuCyte Basic Analyzer software (Version 2021A, Sartorius AG, Göttingen, Germany). Data represent mean  ±  SD of 4 photos x well for at least 3 wells for every treatment condition.

#### 4.8.7. Confocal Microscopy

Cells were seeded on glass-bottom dishes (WillCo-dish^®^, WillCo Wells B.V., Amsterdam, The Netherlands; 30 mm glass diameter) at a density of 7 × 10^4^ cells per dish in complete culture medium and treated with nanoformulations for 2 h. Lysosomes, mitochondria, and nuclei were stained with LysoTracker Green, MitoTracker Deep Red, and Hoechst 33342, respectively. Cells were fixed with 4% paraformaldehyde and imaged using a Leica TCS SP8 (Leica Microsystems GmbH, Wetzlar, Germany) confocal microscope equipped with 405, 488, 561, and 633 nm laser lines. Sequential scanning was used to avoid spectral overlap. Raw confocal image stacks were processed using Huygens Essential software (Version 25.10, Scientific Volume Imaging B.V., Hilversum, The Netherlands) for deconvolution and image restoration.

## 5. Conclusions

In summary, we have developed a multifunctional nanoplatform combining POPC-Rhod liposomes, alverine, and graphene oxide for targeted delivery and fluorescence-guided cellular tracking in neuroblastoma models. The hybrid systems exhibit robust physicochemical features, strong fluorophore–graphene interactions, and efficient cellular internalization. Despite some limitations, the results highlight the potential of graphene oxide–lipid hybrids as adaptable nanocarriers for the delivery of repurposed drugs to neural cancer cells. The combination of efficient fluorescence-based tracking, phenotype-dependent cytotoxicity, and enhanced intracellular delivery supports further exploration of this platform as a candidate system for targeted neuro-oncological nanotherapy.

## Figures and Tables

**Table 1:** Hydrodynamic diameter (Z-average) and polydispersity index (PDI) of liposomes containing different concentrations of alverine measured by dynamic light scattering before and after sonication.

ALV Concentration	Sonication	Z-Average, nm	PDI
0%	✓	282.1	0.570
1%	✓	51.4	0.444
	✖	1.70 × 10^7^	0.670
2.5%	✓	328.3	0.654
5%	✓	165.4	0.403

**Table 2:** Nanomechanical properties of liposomes determined by atomic force microscopy, including vesicle height, adhesion force, deformation, Young’s modulus, stiffness, and energy dissipation for different alverine concentrations and sonication conditions. Values are reported as mean ± SD from at least three independent measurements.

	ALV 0%	ALV 1%	ALV 1%	ALV 5%
Sonication:	✓	✓		✓
Height, nm	52 ± 3	43 ± 7	27 ± 3	40 ± 4
Adhesion energy, aJ	85 ± 5	233 ± 6	411 ± 42	51 ± 8
Adhesion force, nN	8.9 ± 0.2	12.7 ± 0.8	21 ± 3	5.1 ± 0.8
Deformation, nm	10.3 ± 0.3	36 ± 2	11.3 ± 0.7	14.9 ± 0.2
Energy dissipation, fJ	1.5 ± 0.2	1.81 ± 0.03	2.5 ± 0.2	2.6 ± 0.1
Young’s modulus, MPa	620 ± 50	169 ± 14	685 ± 133	694 ± 156
Stiffness, N/m	16.5 ± 0.6	7 ± 1	33 ± 3	19.9 ± 0.8

## Data Availability

The original contributions presented in this study are included in the article and Supplementary Materials. Further inquiries can be directed to the corresponding author.

## Supplementary Materials

The following supporting information can be downloaded at: https://www.mdpi.com/article/10.3390/ijms27073273/s1.

## Abbreviations

The following abbreviations are used in this manuscript:

ROS	Reactive oxygen species
MQ	Milli-Q
usGO	Ultrasonicated graphene oxide
SUV	Small unilamellar vesicle (here specifically, vesicle after extrusion)
SUV-Rhod-ALV	Small unilamellar vesicle of POPC and Rhodamine with ALV added to the chloroform solution; after extrusion
SUV-Rhod+ALV	Small unilamellar vesicle of POPC and Rhodamine with ALV added to PBS during rehydration; after extrusion
POPC-Rhod	Vesicle of POPC and Rhodamine; before extrusion
POPC-Rhod+ALV	Vesicle of POPC and Rhodamine with ALV added to the chloroform solution; before extrusion
