# Supplementary material for: Alverine-Loaded Lipid Bilayer–Graphene Oxide Hybrids as a Novel Nanomedicine Platform for Neural Cancer
Source: Int J Mol Sci. 2026 Apr 4;27(7):3273. doi: 10.3390/ijms27073273 (PMC13072880; doi:10.3390/ijms27073273)
Supplement: Supplementary file 1 [file ijms-27-03273-s001.zip › ijms-4204457-supplementary.pdf]

ESI:

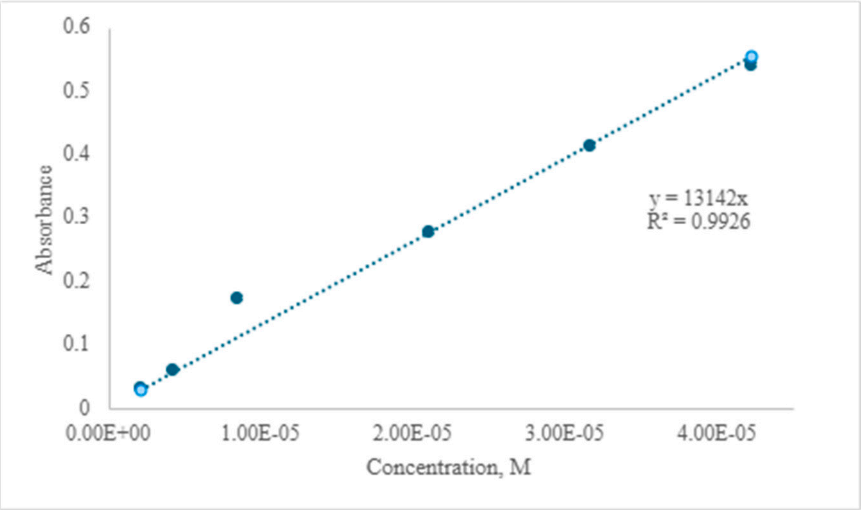

Figure S1. Calibration curve of PE-Rhod in PBS measured at λ=574 nm.

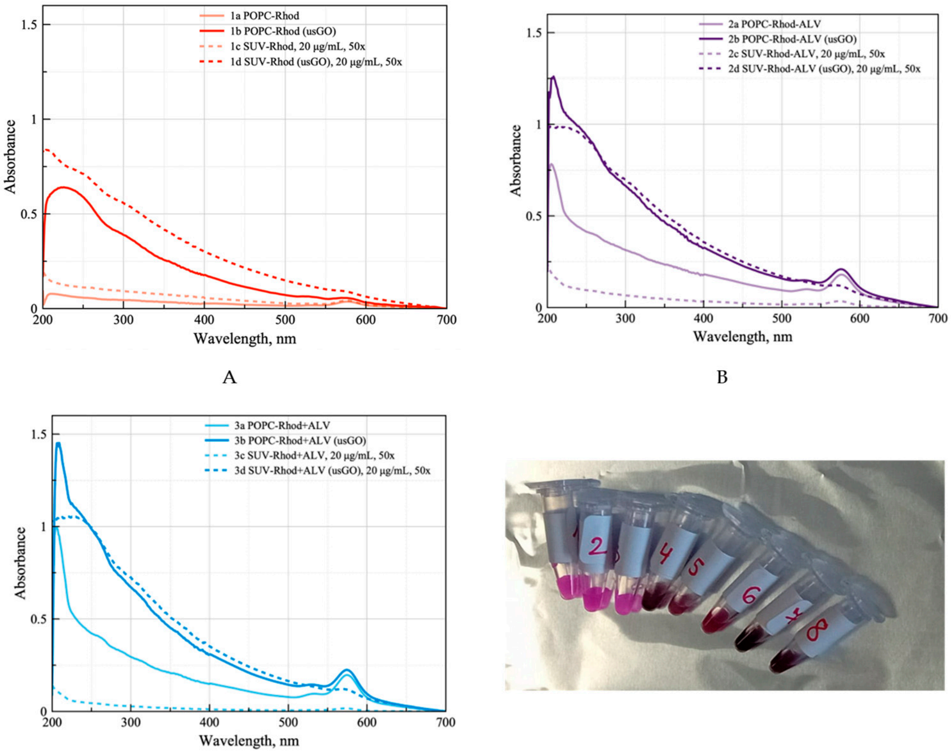

C

D

Figure S2. A comparison of the UV–Vis spectra of the vesicles after extrusion (panels A-C); panel D—photographs of the samples: 1—POPC-Rhod (0.03 μm), 2—POPC-Rhod-ALV (0.03 μm), 3—POPC-Rhod+ALV (0.03 μm), 4—POPC-Rhod in usGO (0.03 μm), 5—POPC-Rhod-ALV in usGO (0.03 μm), 6—POPC-Rhod+ALV in usGO (0.1 μm), 7—POPC-Rhod-ALV in usGO (not extruded), 8—POPC-Rhod+ALV in usGO (not extruded); values in brackets correspond to the size of pores in extrusion membrane.

Concerning the SUV alone (panel A), an increase and broadening of absorption is observed, alongside the loss of clear evidence of the peak and shoulder characteristic of the GO. As per the POPC-Rhod-ALV (panel B) and POPC-Rhod+ALV (panel C), although both samples after the

extrusion do not exhibit the peak at 215 nm anymore, the presence of ALV is still confirmed by the positive difference spectra compared to the GO alone.

Table S1. The concentrations of PE-Rhod in the samples.

SAMPLE	DILUTION (X)	CONCENTRATION	
**1.**	1000	1 μg/ml	$4.22 \cdot 10^{-5}$ M
**2.**	400	2.5 μg/ml	$3.17 \cdot 10^{-5}$ M
**3.**	250	4 μg/ml	$2.11 \cdot 10^{-5}$ M
**4.**	100	10 μg/ml	$8.45 \cdot 10^{-6}$ M
**5.**	66,6	15 μg/ml	$4.22 \cdot 10^{-6}$ M
**6.**	50	20 μg/ml	$2.11 \cdot 10^{-6}$ M

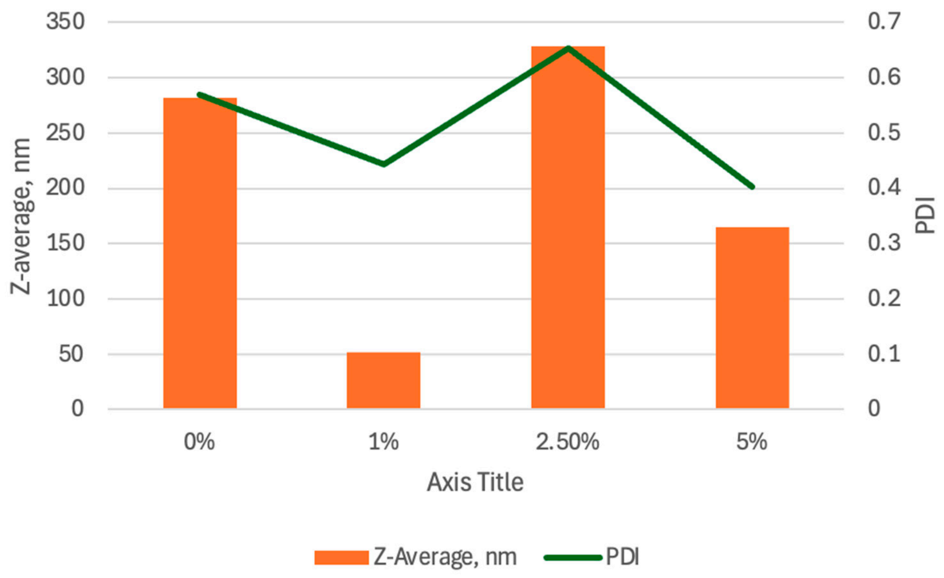

Figure S3. Hydrodynamic diameter (Z-average) and polydispersity index (PDI) of liposomes containing different concentrations of alverine measured by DLS after sonication.

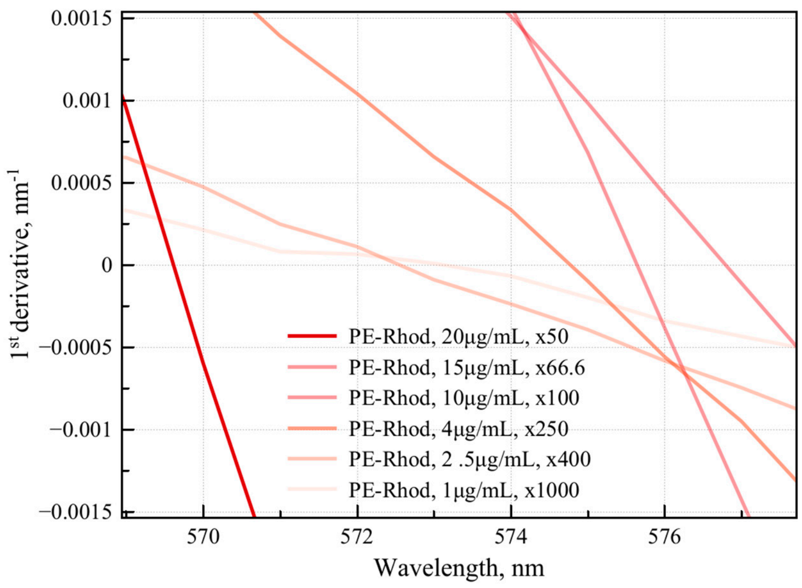

Figure S4. First derivatives of PE-Rhod UV–Vis absorption spectra.

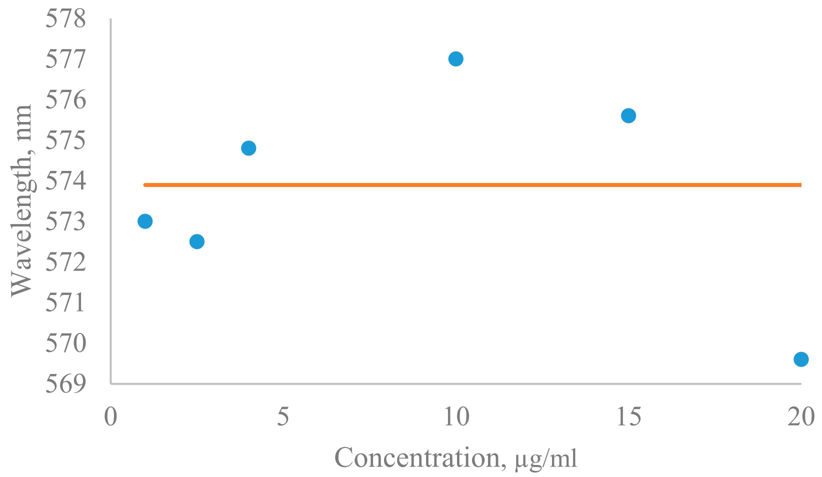

Figure S5. Representation of wavelengths as a function of concentration.

Table S2. List of the substances used for SUV preparation.

SAMPLE	$CHCl_3$ [µl]	POPC [µl]	PE- Rhod [µl]	ALV [mg]
1	700	200	100	-
2	700	200	100	1 (added to $CHCl_3$)
3	700	200	100	1 (added to PBS)

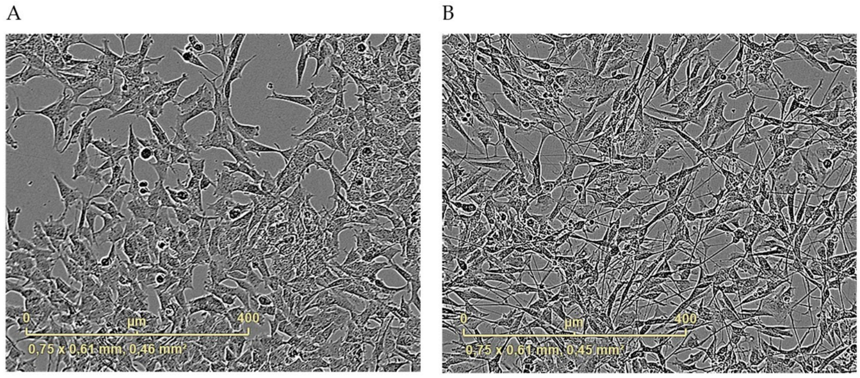

Figure S6. Microscopic images of SH-SY5Y neuroblastoma cells, before differentiation (panel A) and after differentiation (panel B) with retinoic acid.
